# Clinical Lectures upon Surgical Cases in the Buffalo Hospital of the Sisters of Charity

**Published:** 1871-10

**Authors:** J. F. Miner


ART. IV.—*Clinical Lectures upon Surgical Cases in the Buffalo Hospital of the Sisters of Charity.* By PROF. J. F. MINER.

*Reported by Louis Brecht, member of the class of* 1871-72.

GENTLEMEN,—It will be of greater importance and value to you to notice with care the cases which are more apt to present themselves in your every-day practice, and are known under the name of Minor Surgery, than the rarer, and what you might naturally regard as the more interesting capital operations of Surgery. I shall, therefore, take the greatest pleasure in showing you such cases as come constantly under our care, and such as will be your chief occupation as Surgeons. Upon your aptness in the management of such cases as we present this morning will largely depend your future success and reputation as practical Surgeons.

CASE I.—*Enchondroma.* Dr. Niemeyer, of Canada, who is on a visit to us for a few days, has kindly presented you with an interesting pathological specimen called Enchondroma. This is the name applied to a cartilagenous growth, which holds an intermediate place between the fibrous and oseous tumors. These tumors, however, are not alike either in character nor form. They may be gelatinous, and have the appearance of containing matter, or may be hard, smooth and elastic; they may be round or flattened. Their growth is attended with no pain. They occur mostly in young people with a rickety constitution and scrofulous diathisis They may occur in any part of the body; more frequently however on the fingers and toes, and are generally attached to bony walls. The present specimen is the left fore finger, amputated for this affection. After making a section through it, we find it to be homogeneous, consisting of a firm, fibrous, whitish mass. These growths may appear in the testes, mammary or parotid glands in which case they are more liable to undergo degeneration and become malignant. At times, these tumors attain an enormous size. A specimen of this character is mentioned in Gross's Surgery, in which the shoulder and clavicle were involved, weighing thirty-one pounds. They are said to break down occasionally, causing deep cavities. Occasionally the entire system becomes affected, and may have the appearance of encephaloid disease. The treatment may be either extirpation of the tumor, or amputation of the entire member thus affected. The latter is generally resorted to as the disease is less liable to return.

CASE II.—*Cataract.* Mrs. Seely, a private patient of mine, aged 45, has consented to appear before you, that you may observe the result of an operation for cataract. It is now eight weeks since the operation was performed. It was made by what is called Von Graefe's method of linear extraction, modified a little. Both eyes were operated upon at the same time, and I am happy to say, the result is entirely satisfactory. You will notice that the field of vision is entirely clear; that the small incision made for the extraction of the lens at the upper margin of the cornea is but slightly visible and that the iris is a little attached at this point. The patient can read No. 6 of Jaeger's type, by aid (of course) of proper cataract glasses. A few months hence her vision will doubtless be more distinct. The extracted lens in all cases must be replaced by artificial lens, or cataract glasses. Distant objects may be seen more distinctly than near ones but distinct vision of near objects require glasses. They should, however, never be used until at least two or three months after the operation; early and protracted use of the eyes is apt to cause congestion of the retina and is unsafe.

CASE III.—*Inverted Toe Nail.* John McNamee, aged 18, dresser in the hospital, suffers from an inverted nail on his first toe. The nail has been removed before and this is its second growth. It has an unhealthy stubby appearance and has an ulcerating surface underneath and again requires removal. This condition of the nail is of greater importance than it seems; it actually disables the sufferer, often for a longer time than a broken leg, and is very vexatious and painful. The patient is ready to undergo any operation you may propose, even amputation of the toe; the latter, however, is quite too radical for ordinary purposes. Various plans of procedure have been tried to effect a cure, such as Per Sulphate of Iron introduced between the flesh and nail which tans the flesh, so that it will separate, or form a sort of slough, and has been highly spoken of; crowding cotton down by the side of the nail to keep the flesh separated from it; drawing the flesh from the nail by means of adhesive strips, and other methods have been recommended as paliative but have generally proved to be of only temporary value. Extraction is by far the most efficient method of treatment. By throwing on the toe the spray of ether or giving chloroform in the usual way the operation may be easily and painlessly performed. In a few days a scale will form which will answer every purpose of a nail, until the new one is formed. By dressing the toe with a thin, light and dry cloth, so as not to exclude the air from the wound, the formation of the latter will be facilitated.

CASE IV.—*Cartarrhal Conjunctivitis.* We have for treatment this morning four patients with what is called Catarrhal Conjunctivitis or more accurately describing the conditions present, granular lids and vascular cornea. The conjunctiva or mucous covering of the globe and lining of the lids contains in its anatomical structure papillæ, which, when affected by inflammation enlarge and form the large, fine points which you observe as I invert the upper lid of the first patient. Both upper and lower lids are highly inflamed, and the rough surface is very apparent. On the inner portion of the upper lids of this patient is a deposit of lymph making the so-called trachoma. The cornea in both eyes is very hazy and vascular, conditions which are caused by the roughness of the lids. A little below the centre of the cornea in one eye you observe a dark point. This is the site of an ulcer which has extended through the cornea; the iris has fallen against its walls, being carried forward by the escape of the aqueous humor and has there attached itself; the ulceration has healed, and fortunately vision has not been destroyed. You thus see that ulceration and even perforation of the cornea may follow from the effects of this disease, and thus vision may be greatly impaired or even lost.

In the second patient, the disease is somewhat modified. The upper half of the cornea is opague and vascular while the lower half is clear. Thus showing that the disease is confined to the upper lid mainly. In the third patient we find the upper lid also in a red and roughened condition and the cornea but slightly affected. In the fourth patient you will notice only a few enlarged papillæ or granulations while a large portion of the lid appear white. This is a sign of convalescence. The treatment of all these cases is the same. The first object is to prevent extension of the disease in all cases where there is any manifest contagious tendencies. Great cleanliness, and generally good diet is required. Nothing is more common than broth for food, with blisters, leeches and other counter-irritation, and nothing is so sure to end in failure. Various collyria have been depended upon, most of them of little use. The crayon of nit. silver, or a saturated solution applied to the rough surface might be efficient, but there are serious objections to its continued use, which I will hereafter explain. The safest and most efficient treatment ever yet proposed, is to apply he crayon of sul. copper to the surfaces of both upper and lower lids, daily. If you will adopt this and adhere to it, you will be *always* successful. If perseveringly and carefully applied, it is certain and uniformly satisfactory.

